# Progress in Promising Semiconductor Materials for Efficient Photoelectrocatalytic Hydrogen Production

**DOI:** 10.3390/molecules29020289

**Published:** 2024-01-05

**Authors:** Weisong Fu, Yan Zhang, Xi Zhang, Hui Yang, Ruihao Xie, Shaoan Zhang, Yang Lv, Liangbin Xiong

**Affiliations:** 1 School of Optoelectronic Engineering, Guangdong Polytechnic Normal University, Guangzhou 510665, China; fwsdsg_1995@163.com (W.F.); zhangyanlsdw@outlook.com (Y.Z.); zhangxi07222023@163.com (X.Z.); xieruihao@gpnu.edu.cn (R.X.); zsagdut@yeah.net (S.Z.); lvyang@gpnu.edu.cn (Y.L.); 2 School of Medical Information Engineering, Gannan Medical University, Ganzhou 341004, China

**Keywords:** photoelectrocatalysis, hydrogen energy, semiconductor, photoelectrode

## Abstract

Photoelectrocatalytic (PEC) water decomposition provides a promising method for converting solar energy into green hydrogen energy. Indeed, significant advances and improvements have been made in various fundamental aspects for cutting-edge applications, such as water splitting and hydrogen production. However, the fairly low PEC efficiency of water decomposition by a semiconductor photoelectrode and photocorrosion seriously restrict the practical application of photoelectrochemistry. In this review, the mechanisms of PEC water decomposition are first introduced to provide a solid understanding of the PEC process and ensure that this review is accessible to a wide range of readers. Afterwards, notable achievements to date are outlined, and unique approaches involving promising semiconductor materials for efficient PEC hydrogen production, including metal oxide, sulfide, and graphite-phase carbon nitride, are described. Finally, four strategies which can effectively improve the hydrogen production rate—morphological control, doping, heterojunction, and surface modification—are discussed.

## 1. Introduction

Hydrogen is known as the 21st century’s most promising clean energy due to its status as an efficient energy carrier with a high energy combustion value (142.5 kJ/g), and the end product of its use is water [1,2]. To some extent, the development and utilization of hydrogen energy in relation to renewable energy sources has alleviated the fossil energy crisis [3,4]. Solar energy is inexhaustible but inconveniently used due to its dispersion. In this case, converting solar energy into hydrogen energy is a very promising energy solution because it can be more easily stored and effectively used [5,6].

Photoelectrocatalytic (PEC) hydrogen evolution using semiconductor photoelectrodes under irradiation has been widely studied as one of the effective ways to convert solar energy into hydrogen energy [7,8]. TiO_2_ has been one of the most important and frequently studied PEC photoelectrodes since the discovery of the Fujishima–Honda effect [9]. After decades of development, PEC photoelectrodes have sufficiently expanded to a variety of TiO_2_-based and other new photoelectrode materials; the influence of these photoelectrodes on PEC hydrogen production has been widely studied, and the related mechanisms have been exhaustively discussed [10,11,12].

The purpose of this review is to consider the progress on photoelectrocatalysis (PECs) for hydrogen generation from water using high-performance photoelectrode materials. In particular, this work considers the key material properties of photoelectrodes and the trends in research focused on the development of photoelectrodes that may bring PEC technology to commercial maturity. We focus on the preparation and modification of semiconductor photoelectrode materials that have been widely investigated by researchers. Various high-performance photoelectrode materials, including metal oxide, sulfide, and graphitic carbon nitride semiconductors, are discussed. Numerous considerations are limited solely to oxide materials, which appear to exhibit superior properties as photoelectrodes in comparison to other types of materials.

## 2. The PEC Process of Water Decomposition

### 2.1. Mechanisms

PECs combines the advantages of both photocatalytic and electrocatalytic technologies. PECs can both make use of solar energy and regulate the photocatalytic process by using photoelectrodes with an appropriate external bias. Moreover, photoelectrodes are convenient for recycling in PEC processes, avoiding the dilemma encountered in the photocatalytic process.

When a semiconductor (e.g., photoanode) is irradiated, electrons and holes can be formed in the conduction band and the valence band, respectively. An electric field at the electrode/electrolyte interface is required in order to avoid the recombination of these charge carriers. The electrons generated in the photoanode are transferred over the external circuit to the cathode, resulting in a reduction reaction (for example, hydrogen ions are reduced into gaseous hydrogen). The light-induced holes lead to the splitting of water molecules into gaseous oxygen and hydrogen ions. The overall reaction for the PEC process may be expressed in the following form:

(1) $$2h\nu +H_{2}O\to \frac{1}{2}O_{2}(gas)+H_{2}(gas).$$

Reaction (1) takes place through a high energy barrier with a Gibbs free energy value of 237.2 kJ/mol, and the electrochemical decomposition of water is possible when the electromotive force of the cell is equal to or larger than 1.23 eV [13].

**Figure 1:**
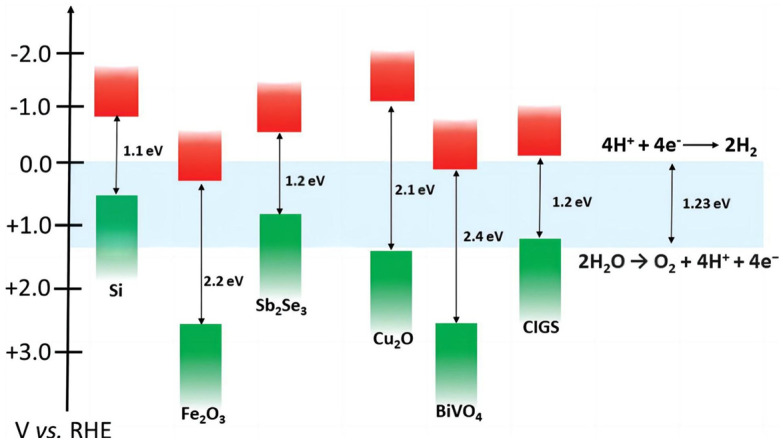
Band structures of typical semiconductors [16]. Reproduced with permission from Yang, W., *Chemical Society Reviews*, published by Royal Society of Chemistry, 2019.

Concerning the mechanisms of the reactions, the principle of PEC water decomposition is highly related to the energy band structure of the photoelectrode semiconductor. Figure 1 shows a schematic diagram of a typical semiconductor energy band structure, illustrating the various band levels of established electrodes and comparing them with the potentials corresponding to H_2_/H^+^ and H_2_O/O_2_ redox couples. The bandgap of the photoelectrode semiconductor should be greater than 1.23 eV for the overall reaction for PEC water decomposition. Meanwhile, the conduction band of the semiconductor should be more negative than the electrode potential of H_2_/H^+^, while the valence band position should be more positive than the electrode potential of H_2_O/O_2_ for the complete decomposition of water. Moreover, the water decomposition voltage is frequently higher than the theoretical value of 1.23 V [14] due to the existence of overpotential in practical PEC systems. It is well known that most semiconductors can satisfy only one of these conditions [15]. Figure 1 shows the band structures of some semiconductors.

In addition, the hydrogen half-reaction involves the transfer of only two electrons, whereas the half-reaction of oxygen production requires the transfer of four electrons and the formation of O-O bonds. Therefore, the generation of hydrogen is thermodynamically favorable, and the oxygenation reaction is the bottleneck of the whole reaction process if one takes into account the slowest process that determines the overall rate [17].

### 2.2. PEC Cell

Generally, the PEC cell is composed of a photoelectrode, counter electrode, reference electrode, and electrolyte solution. The photoelectrode, used as the working electrode, is mainly composed of a conductive substrate and a semiconductor film material. N- and p-type semiconductors are generally used as photoanodes for PEC oxygen production and photocathodes for PEC hydrogen evolution, respectively.

Materials with good charge collection performance and low reaction overpotential, such as platinum counter electrodes, are usually selected to fulfil the role of the counter electrode.

The electrolyte solution is responsible for mass transfer in the PEC cell. Therefore, the electrolyte solution should have a high conductivity. Salt substances such as Na_2_SO_4_ and K_2_SO_4_ are commonly used in neutral electrolyte solution, while acidic and alkaline solutions, as well as buffer solution, are employed for specific experimental conditions.

**Figure 2:**
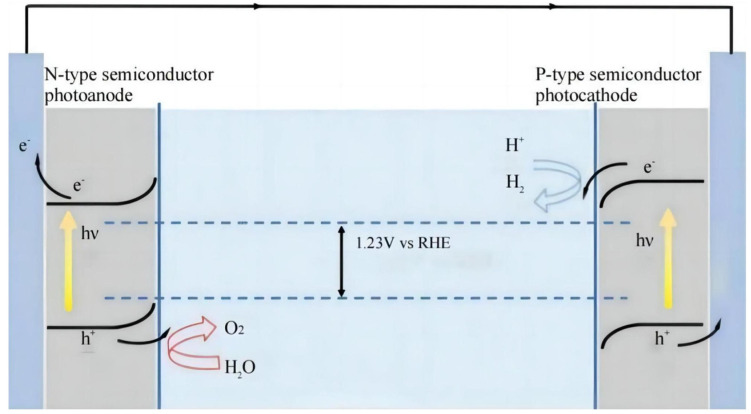
Diagram of a PEC tandem cell [20]. Reproduced with permission from Grätzel, M., *Nature*, published by Springer Nature, 2001.

PEC cells can be divided into PEC photoanode cells, PEC photocathode cells, and PEC tandem cells, depending on the way that the semiconductor photoelectrodes are arranged. The PEC photoanode cell generally takes a n-type semiconductor as the working electrode and a platinum electrode as the counter electrode. The PEC photocathode cell often uses a p-type semiconductor as the working electrode and a platinum electrode as the counter electrode. The configuration of the PEC tandem cell is depicted in Figure 2, where a n-type and a p-type semiconductor are used as a photoanode and photocathode, respectively. The first two kinds of cells, using a single semiconductor as a working electrode, frequently require an external bias to promote the decomposition of water [16]. Fortunately, in a PEC tandem cell, the combination of a photoanode and photocathode is highly possible to ensure the complete photosplitting of water under sunlight without bias. As shown in Figure 2, photoanode and photocathode materials with different light absorption ranges can broaden the spectral range in the PEC tandem cell. The configuration of the PEC tandem cell is helpful for increasing photocurrent density and improving the efficiency of solar water splitting [18,19].

## 3. Semiconductor Materials for PECs

In 1972, Fujishima Akira et al. [9] first reported the successful PEC decomposition of water on the surface of TiO_2_ single-crystal electrodes under UV irradiation. Subsequently, researchers around the world observed a similar phenomenon of PEC hydrogen production on other semiconductor materials, such as BiVO_4_ [21,22], Cu_2_O [23,24], Fe_2_O_3_ [25,26], WO_3_ [27,28], and ZnO [29,30]. After decades of development, PEC hydrogen evolution using semiconductor photoelectrodes has made great progress.

### 3.1. Metal Oxide Materials

Currently, metal oxide semiconductors are considered for their advantages in terms of photochemical stability, their low cost, and their suitability for large-scale preparation [31,32,33]. TiO_2_ has been widely studied due to its excellent photocatalytic activity, stability, safety, non-toxicity, rich content, and ease of preparation. However, TiO_2_ with a wide bandgap of 3.2 eV can only be activated by UV light with a wavelength below 387 nm. Thus, many efforts have been taken to improve the PEC efficiency of TiO_2_. For example, the visible light activity of TiO_2_ can be enhanced by modification. In particular, in the late 1980s, researchers devoted their efforts to developing second-generation TiO_2_ that could absorb both UV (290–400 nm) and visible (400–700 nm) light and thereby enhance the overall efficiency. N-type BiVO_4_ [34] and p-type Cu_2_O [35] are widely regarded as promising semiconductor materials for efficient PEC hydrogen production due to their bandgap widths and suitable band structures.

#### 3.1.1. TiO_2_

TiO_2_, possessing excellent photoelectric performance and high photochemical stability, has always been a very promising PEC material. However, as mentioned above, TiO_2_ can only absorb UV light, and the photogenerated charges are easy to recombine, resulting in a low PEC efficiency. Fortunately, the PEC efficiency of TiO_2_ can be effectively improved by doping and constructing composite structures.

(1) Doping

**Figure 3:**
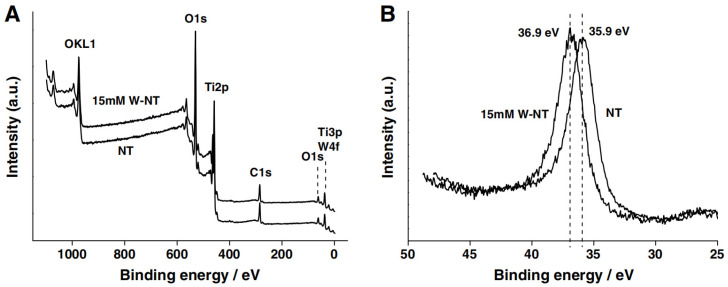
XPS spectra of (**A**) global range and (**B**) high-resolution spectra of Ti*3p* orbit of TiO_2_NTs and 15 mM W-TiO_2_NTs, respectively [36]. Reproduced with permission from Gong, J., *Catalysis Communications*, published by Elsevier, 2013.

**Figure 4:**
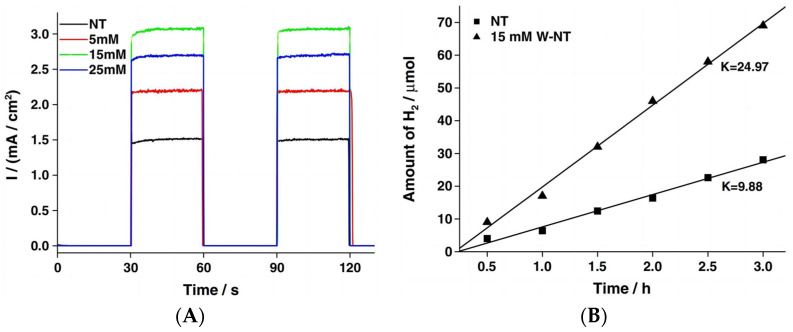
(**A**) The transient photocurrent responses of W-TiO_2_ NTs. (**B**) Amount of H_2_ evolution in the photocatalytic processes of TiO_2_ NTs and 15 mM W-TiO_2_ NTs, respectively [36]. Reproduced with permission from Gong, J., *Catalysis Communications*, published by Elsevier, 2013.

Gong [36] and co-workers reported a tungsten-doped TiO_2_ nanotube arrays (W-TiO_2_ NTs) photoelectrode with an exclusive anatase phase utilizing a facile and novel anodization process on a Ti sheet. The results showed that W atoms were successfully incorporated into the TiO_2_ lattice in the form of W^6+^ ions, which did not influence the morphologies of the TiO_2_ NTs samples. The samples were evaluated by XPS to analyze the chemical states of W in W-TiO_2_ NTs. The peaks of C*1s*, O*1s*, Ti*3p*, Ti*2p*, and W*4f* were observed, as shown in Figure 3A. Figure 3B clearly shows that the peak of Ti*3p* shifted from 35.9 to 36.9 eV because of the presence of W in the TiO_2_ lattice. Thus, the W^6+^ ions were loaded into the bulk TiO_2_ lattice by displacing Ti^4+^ ions and forming W-O-Ti bonds. As shown in Figure 4, the highest photocurrent density and hydrogen production rate were observed for the 15 mM W-TiO_2_ NTs annealed at 400 °C. Under illumination, the photocurrent density quickly reached a constant value of 0.3 mA/cm^2^, which indicates that the transfer of the photogenerated charge is quite rapid. The photocurrent pattern is highly reproducible for several on–off light cycles. The photogenerated electrons are rapidly transported from the TiO_2_ nanotube arrays to the counter electrode to produce the photocurrent [37].

**Figure 5:**
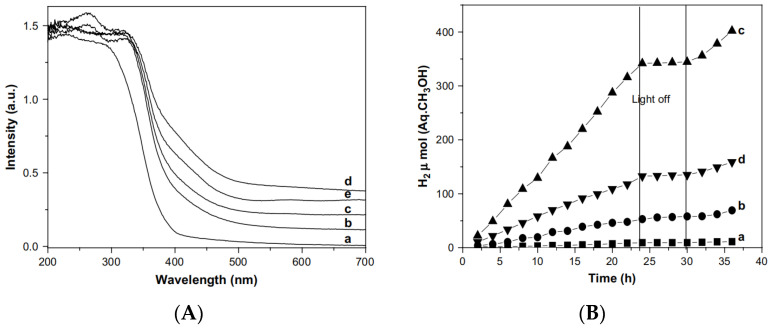
(**A**) UV-vis DRS spectra of (a) TiO_2_, (b) 0.1 wt% Fe/TiO_2_, (c) 0.5 wt% Fe/TiO_2_, (d) 1.0 wt% Fe/TiO_2_, and (e) 2.0 wt% Fe/TiO_2_. (**B**) Hydrogen evolution in photocatalytic water splitting in 20% methanol aqueous solution under visible light irradiation: (a) 0.1 wt% Fe/TiO_2_, (b) 0.5 wt% Fe/TiO_2_, (c) 1.0 wt% Fe/TiO_2_, and (d) 2.0 wt% Fe/TiO_2_ [41]. Reproduced with permission from Khan, M. A., *International Journal of Hydrogen Energy*, published by Elsevier, 2008.

The PEC performance of TiO_2_ can also be improved by metal element doping to increase its conductivity. A simple saturated aqueous solution method was used to synthesize a novel rutile Nb-TiO_2_/g-C_3_N_4_ photoanode, whose photocurrent was 1.39 times larger than that of its pristine counterparts under UV light [38]. In addition, the unoccupied conduction band of TiO_2_ comprises Ti*3d*, *4s*, *4p* orbitals, whereas the occupied valence band (VB) comprises O*2p* orbitals. Ti*3d* orbitals dominate in the lower position of the conduction band [39,40]. An impurity level can be induced by doping with other metal ions (cations) in place of Ti. The resultant intermediate energy level promotes visible light absorption by acting as either an electron donor or acceptor. By doping Fe into TiO_2_, the absorption peak of the sample can gradually shift to red with the increase in the amount of Fe^3+^ loading, as shown in Figure 5A. The red shift of the absorption edge in Fe/TiO_2_ might be attributed to the excitation of *3d* electrons of Fe^3+^ ions to the TiO_2_ conduction band (charge transfer transition) [41]. Figure 5B shows that the hydrogen evolution rate reached the maximum in the 1.0 wt% Fe/TiO_2_ photocatalyst. Meanwhile, the H_2_ evolution rate decreased in the 2.0 wt% Fe/TiO_2_ photocatalyst, which might be due to the role of excess Fe^3+^ as a recombination site. Compared with the W-TiO_2_ NTs mentioned above, the hydrogen production rate was reduced because more energetic ultraviolet light below 400 nm is shielded. However, its response to visible light makes Fe/TiO_2_ more widely used.

(2) TiO_2_-based composites

**Figure 6:**
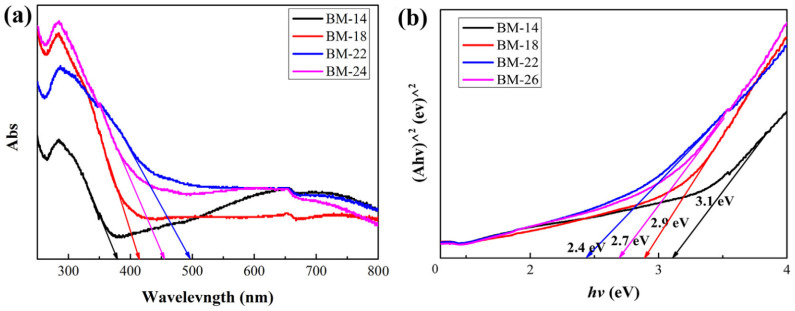
UV-Vis absorption (**a**) and a Kubelka–Munk plot (**b**) of a TiO_2_ NTs/Bi_2_MoO_6_ heterojunction photocatalyst [42]. Reproduced with permission from Liu, Z., *Journal of Colloid and Interface Science*, published by Elsevier, 2019.

**Figure 7:**
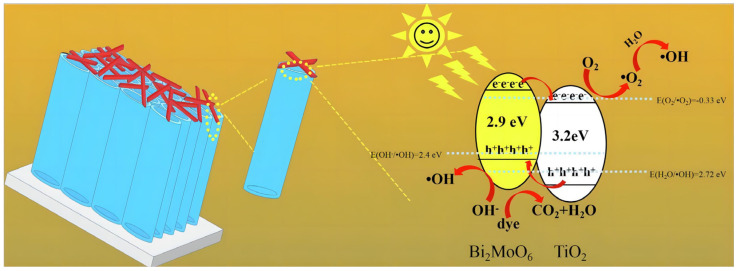
Schematic diagram of the degradation of dyes by a TiO_2_ NTs/Bi_2_MoO_6_ heterojunction photocatalyst under solar irradiation [42]. Reproduced with permission from Liu, Z., *Journal of Colloid and Interface Science*, published by Elsevier, 2019.

Liu [42] and co-workers reported a TiO_2_ NTs/Bi_2_MoO_6_ type-II heterojunction (a detailed description of type-II heterojunctions is available in Section 3.3) photocatalyst prepared using a simple solvothermal method. Bi_2_MoO_6_ nanoparticles (NPs) with nanosheet microstructures were successfully loaded on the surface of TiO_2_ NTs through the adjustment of reaction intervals. With increasing reaction time, the amount of Bi_2_MoO_6_ deposition increased gradually. The absorption edges of the samples with reaction times of 14, 18, 22, and 26 h were located at 380, 414, 495, and 452 nm, respectively (Figure 6a). Furthermore, the band gaps of the samples could be estimated using transformational Tauc plots, and their band gaps were calculated to be 3.2, 2.9, 2.4, and 2.7 Ev (Figure 6b). Reductions in band gap values were beneficial to solar energy absorption and photoelectric performance. Because the CB and VB position of Bi_2_MoO_6_ are more negative than TiO_2_, a type-II heterojunction can be formed at the interface of TiO_2_ NTs/Bi_2_MoO_6_. As shown in Figure 7, the electrons in the VB of the composite material are motivated by photon energy and jump to the CB. Then, electrons in the Bi_2_MoO_6_ CB transfer to that of TiO_2_, and the holes in the TiO_2_ VB transfer to the Bi_2_MoO_6_ VB via the assistance of the internal electric field of the type-II heterojunction. This structure greatly suppresses the recombination of photogenerated electron–hole pairs.

**Figure 8:**
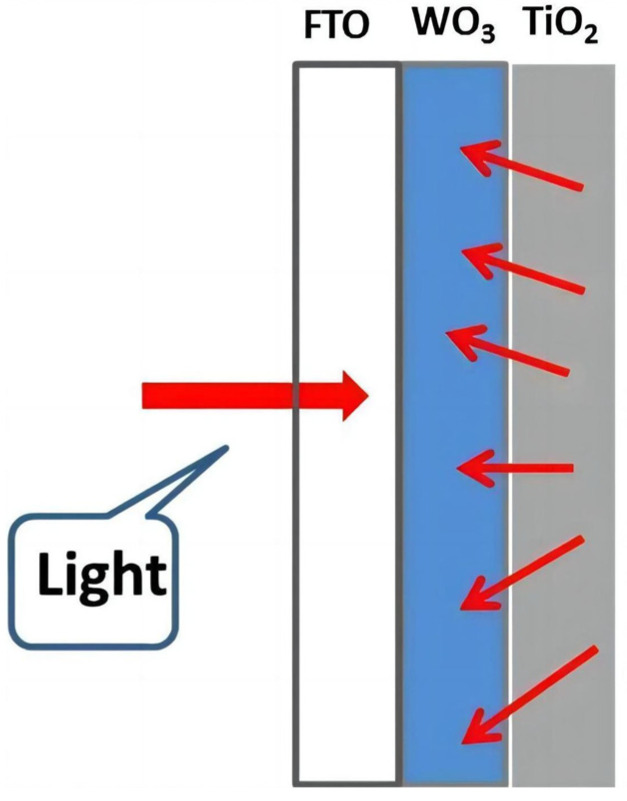
Graphical representation of the direction of photons in the presently used setup [43]. Reproduced with permission from Adamopoulos, P.M., *Catalysts*, published by MDPI, 2019.

Adamopoulos [43] and co-workers reported a photoanode which was made by depositing, on FTO electrodes, either a nanoparticulate WO_3_ film alone or a bilayer film made of nanoparticulate WO_3_ at the bottom, covered with a nanoparticulate TiO_2_ film on the top. Due to the scattering of light by the top TiO_2_ layer, the photocurrent increased by enhancing the light absorption by WO_3_, as shown in Figure 8.

**Figure 9:**
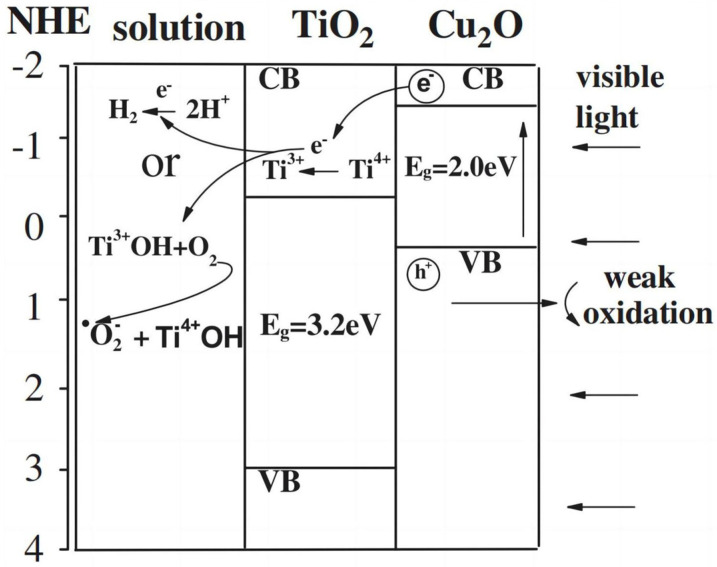
Electron migration diagram of a Cu_2_O/TiO_2_ composite [50]. Reproduced with permission from Xiong, L., *Journal of Physics and Chemistry of Solids*, published by Elsevier, 2011.

Our research group and a few others developed several TiO_2_-based composite systems for energy storage, such as TiO_2_/WO_3_ [44,45], TiO_2_/MoO_3_ [46], and TiO_2_/Ni(OH)_2_ [47]. The energy can be stored either in reduced WO_3_, MoO_3_, or in oxidized Ni(OH)_2_ under UV-light irradiation. In 2008, Yasomanee et al. [48] reported that a TiO_2_/Cu_2_O film photoelectrode led to the continuous generation of H_2_ from water splitting in the dark after UV–vis light irradiation stopped. The following year, we demonstrated that Ti^3+^ in a TiO_2_/Cu_2_O bilayer film has energy storage ability under visible light irradiation. We observed that H_2_ evolution was still noticeable after the irradiation stopped until the third hour. We believe that the electrons trapped in Ti^3+^ ions as stored energy lead to the evolution of H_2_ from H_2_O in the dark [49]. As shown in Figure 9, the photoelectrons of Cu_2_O were captured by Ti^4+^ ions in TiO_2_, resulting in the reduction of Ti^4+^ ions to Ti^3+^ ions. The electrons trapped in Ti^3+^ ions would have been released if suitable electron acceptors existed. Two years later, we [50] also found that the TiO_2_/Cu_2_O composite is capable of both organic degradation and photocatalytic H_2_ evolution under visible light. Different from the two TiO_2_ composites mentioned above, the TiO_2_ layer here also plays a protective role for the Cu_2_O layer. It is well known that Cu_2_O is highly susceptible to photocorrosion. The existence of the TiO_2_ protective layer improves the stability of the photocatalyst.

#### 3.1.2. BiVO_4_

BiVO_4_ is considered as one of the most promising materials in the field of PEC water decomposition thanks to its visible light response, good stability, safety, and non-toxicity. In 1998, Kudo et al. [51] first reported that BiVO_4_ powder can be used as a photocatalyst to decompose water under visible light. Five years later, Sayama et al. [52] first demonstrated that a BiVO_4_-based photoanode successfully decomposed water into hydrogen and oxygen gas.

(1) Doping

At present, BiVO_4_ photoanodes are mainly prepared by coating and electrodeposition. Firstly, a precursor solution containing Bi, V, or other impurity elements are prepared for the deposition of BiVO_4_ films; secondly, the precursor solution is coated on the conductive substrate (FTO or ITO) by spin-coating, dip-coating, spraying, or scraping technology; finally, BiVO_4_ film photoanodes can be obtained by post-annealing [53,54]. The preparation process is convenient for regulating the composition of the film, contributing to a suitable way to study the effect of doping on a BiVO_4_ film. Luo et al. [55] examined the PEC performance of BiVO_4_ photoanodes doped with 11 different kinds of elements through the above process. They found that the PEC performances of the BiVO_4_ photoanodes significantly improved after they were doped with W or Mo. Abdi et al. [56] found that the charge separation efficiency values of the BiVO_4_ photoelectrodes significantly improved as the content of W in the precursor ranged from 0% to 1%.

Kim [57] and co-workers used dimethyl sulfoxide with low hydrophilicity to dissolve acetylace to phenoxy vanadium as a vanadium source solution before coating it on the surface of BiOI thin film electrodes. The problem regarding the poor infiltration of ammonia solution and the BiOI film was solved by this step. Crucially, Kim et al. subjected nanoporous BiVO_4_ to a mild annealing treatment under N_2_ flow, which resulted in nitrogen being incorporated into the oxygen sites. The bandgap of the sample was reduced, while the carrier mobility was increased.

(2) BiVO_4_-based composites

**Figure 10:**
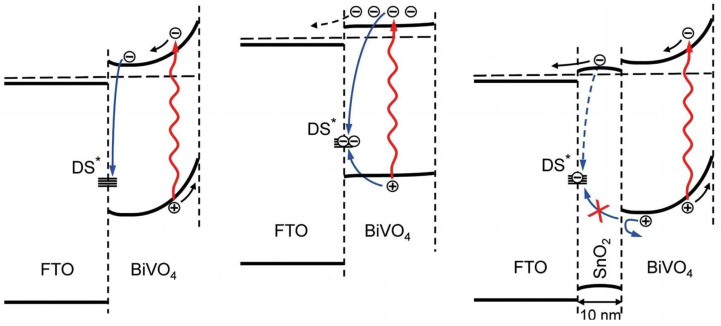
Schematic diagrams illustrating the recombination at the defect state present at the FTO/BiVO_4_ interface and the hole mirror effect of the SnO_2_ layer, respectively [58]. Reproduced with permission from Liang, Y., *The Journal of Physical Chemistry C*, published by American Chemical Society, 2011.

In addition to doping, the separation of photogenerated electron–hole pairs can be promoted by constructing BiVO_4_ heterojunctions to improve PEC performance. Liang et al. [58] reported on highly efficient and reproducible BiVO_4_ photoanodes prepared by a new spray pyrolysis method. As shown in Figure 10, the collection and transfer of carrier charge was substantially improved due to the blocking effect induced on holes, attributable to SnO_2_ being introduced as a thin interfacial layer between the FTO and BiVO_4_. The strategy of using a thin SnO_2_ layer as a hole mirror can benefit other photoanode materials to avoid the recombination of defect states at the FTO/BiVO_4_ interface. This provides an effective method for enhancing the hydrogen production efficiency of FTO-based photoelectrode materials.

**Figure 11:**
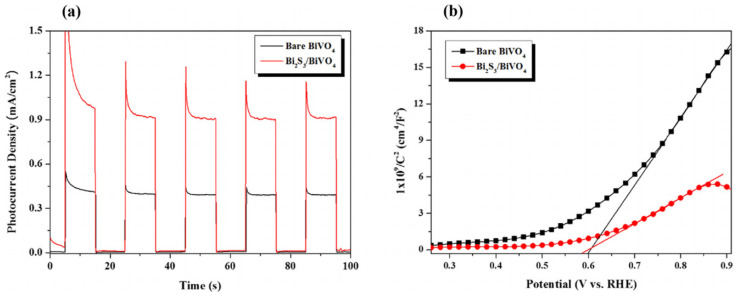
(**a**) Photocurrent density and (**b**) Mott–Schottky plots of the as-prepared photoelectrodes measured in a 0.2 M Na_2_SO_3_ solution [59]. Reproduced with permission from Li, F., *Chemical Engineering Science*, published by Elsevier, 2020.

**Figure 12:**
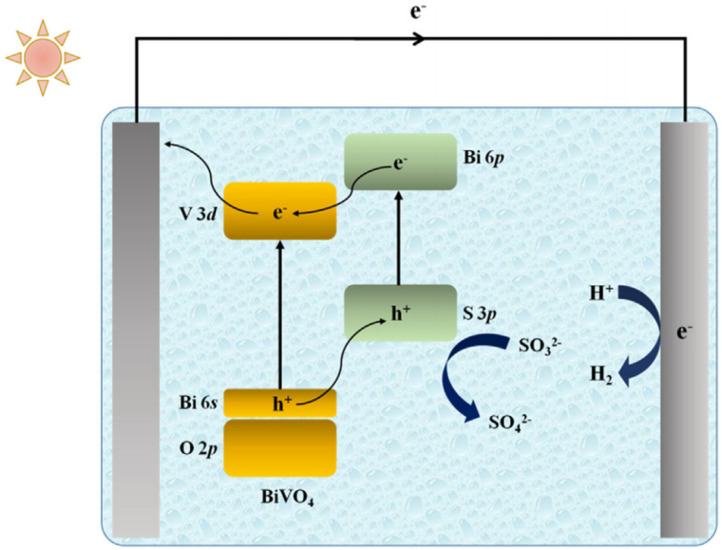
Energy band structure of a hybrid Bi_2_S_3_/BiVO_4_ photoelectrode and its electron transfer pathway in photoelectrocatalytic hydrogen production under solar light irradiation [59]. Reproduced with permission from Li, F., *Chemical Engineering Science*, published by Elsevier, 2020.

Li et al. [59] synthesized a Bi_2_S_3_/BiVO_4_ photoelectrode with a heterojunction structure through a two-step conversion process. As shown in Figure 11a, it is obvious that the hybrid Bi_2_S_3_/BiVO_4_ nanostructure exhibited a higher photocurrent density than the bare BiVO_4_. Compared with Figure 4A, the photocurrent density curve has a sharp decrease at the beginning of each cycle. This phenomenon is caused by the recombination of partial photogenerated charges in the composite, which is very common in composites with heterojunction structures [42,43]. Meanwhile, the results showed that Bi_2_S_3_/BiVO_4_ has a higher carrier concentration relative to the bare BiVO_4_. In addition, the flat band potential had a negative shift for the hybrid Bi_2_S_3_/BiVO_4_ compared with the bare BiVO_4_, which was ascribed to the more negative position of the conduction band for Bi_2_S_3_ than that of BiVO_4_ (Figure 11b). The negative flat band potential shift contributed to the separation of the photogenerated charge. Because the energy band positions of Bi_2_S_3_ were both more negative than that of BiVO_4_, the photoelectrons in the conduction band of Bi_2_S_3_ transferred to the conduction band of BiVO_4_, and the photogenerated holes transferred from BiVO_4_ to Bi_2_S_3_ in the same way (Figure 12). The photocharge recombination can be effectively suppressed, which improves the photocurrent density.

#### 3.1.3. Cu_2_O

Cu_2_O has attracted considerable interest in the field of PECs since Kondo et al. [60]. first reported that Cu_2_O can photocatalytically carry out complete water decomposition in 1998. Cu_2_O has a narrow band gap of about 2.1 eV and a high absorption coefficient. It shows red, orange, and other different colors due to its different synthesis methods and particle sizes. Cu_2_O has various advantages, such as its appropriate band gap, excellent photoelectric performance, simple preparation, and low cost. However, Cu_2_O photocathodes are extremely prone to photocorrosion due to their oxidation–reduction potentials located in the band gap of Cu_2_O [61,62]. At present, a large number of studies in the literature are devoted to the stability of Cu_2_O as a photocathode in the process of PECs. Siripala et al. [63] first proposed that the stability of a Cu_2_O photocathode can be substantially improved if a TiO_2_ protective layer with a thickness of about 100 nm is deposited on the surface of Cu_2_O by the electron beam method.

**Figure 13:**
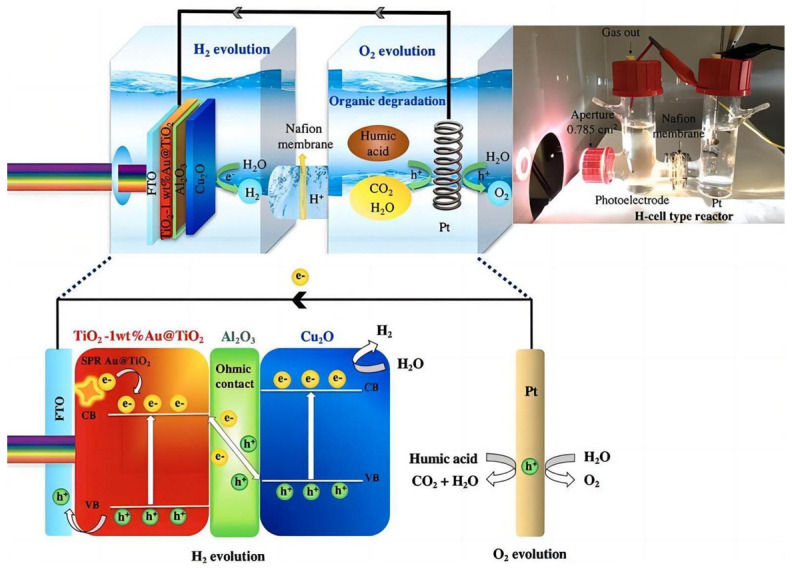
A schematic visualization of a bifunctional PEC system (hydrogen generation and humic acid degradation) with a direct Z-scheme mechanism of TiO_2_-1 wt% Au@TiO_2_/Al_2_O_3_/Cu_2_O photoelectrodes in a H-cell type reactor [65]. Reproduced with permission from Peerakiatkhajohn, P., *Journal of Hazardous Materials*, published by Elsevier, 2021.

Wu et al. [64] reported a MoS_2_/Cu_2_O nanohybrid prepared by a simple wet chemical method. This heterojunction structure effectively promoted the separation of photogenerated electron–hole pairs, resulting in a significant increase in the photocurrent of the nanohybrid. Peerakiatkhajohn et al. [65] originally introduced a Al_2_O_3_ thin-film layer between Au@TiO_2_ and Cu_2_O. On the one hand, strong inherent electric fields at the interfaces, produced by the introduction of an extremely thin Al_2_O_3_ film, suppress the recombination of photogenerated electron–hole pairs. On the other hand, humic acid served as an electron donor to clear holes and suppressed electron hole recombination by capturing the remaining holes. In the presence of humic acid, the hydrogen production efficiency of the PEC device was significantly improved. A bifunctional PEC system with the function of simultaneous hydrogen production and humic degradation was achieved through using the multi-layer Au@TiO_2_/Al_2_O_3_/Cu_2_O photoelectrodes, as shown in Figure 13.

### 3.2. Sulfide Materials

Metal sulfides such as cadmium sulfide (CdS), cadmium zinc sulfide solid solution (Zn_x_Cd_1−x_S), and indium zinc sulfide (ZnIn_2_S_4_) have been extensively studied for their wide range of sources, simple preparation methods, appropriate band structures, and good PEC activity. Solid-phase synthesis is one of the important methods for preparing sulfides. Bao et al. [66] prepared CdS nanocrystals via the pyrolysis of a cadmium thiourea complex at high temperature in a N_2_ atmosphere. However, the crystal produced by this method had a large crystalline grain size and small specific surface area, which is not conducive to the PEC reaction.

With the development of colloidal chemistry, people have gradually developed a variety of liquid-phase synthesis methods to prepare sulfide materials with high PEC performance. In early studies, CdS was often prepared by a chemical precipitation method, and the precursor was usually cadmium brine solution. H_2_S, Na_2_S, thiourea, and other sulfur-containing organic or inorganic compounds are used as precipitants [67]. The microwave hydrothermal method is also an important synthesis method for preparing high-quality sulfides. The electromagnetic field in the microwave reactor changes direction at a frequency of tens of thousands of hertz, causing the dipolar vibration of reactant molecules. The entire reaction system is heated rapidly and evenly, which greatly improves the reaction rate [68].

#### 3.2.1. Doping

**Figure 14:**
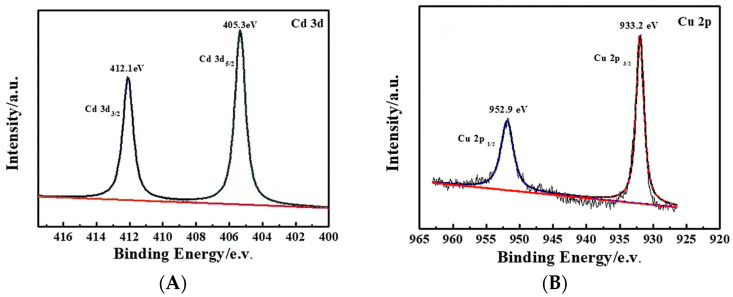
XPS spectra of (**A**) Cd*3d* and (**B**) Cu*2p* [69]. Reproduced with permission from Tian, H., *ChemElectroChem*, published by John Wiley and Sons, 2018.

**Figure 15:**
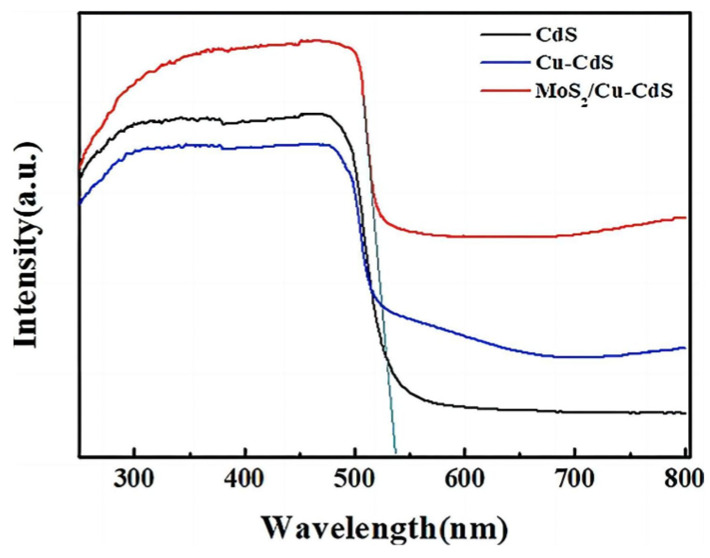
UV/Vis absorption spectra of CdS, Cu-CdS, and MoS_2_/Cu-CdS [69]. Reproduced with permission from Tian, H., *ChemElectroChem*, published by John Wiley and Sons, 2018.

**Figure 16:**
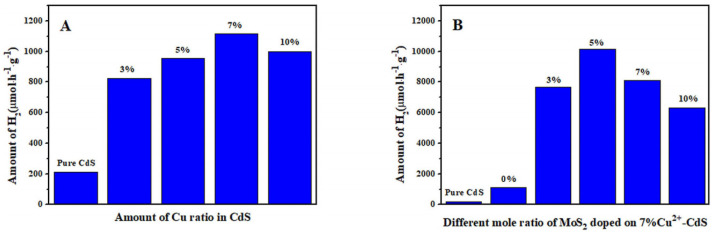
Comparison of the photoelectrocatalytic H_2_ evolution rate of (**A**) Cu-CdS and (**B**) Cu-CdS/MoS_2_ [69]. Reproduced with permission from Tian, H., *ChemElectroChem*, published by John Wiley and Sons, 2018.

For sulfide semiconductors, doping is also an effective way to adjust their band structures for improving PEC performance. Tian et al. [69], Zhang et al. [70], and Barpuzary et al. [71], respectively, incorporated Cu, Co, and Mn elements into CdS, which introduces shallow energy levels into its band gap, improving the PEC performance of doped CdS due to the enhancement in carrier separation efficiency and visible light absorption. As shown in Figure 14, compared with the XPS spectrum of the CdS, the two peaks corresponding to Cd*3d*_5/2_ (404.1 eV) and Cd*3d*_3/2_ (411.7 eV) had a slight shift towards to a higher binding energy. The introduction of Cu^2+^ changed the crystal structure of CdS due to the introduction of lattice defects. Similarly, the absorption edge of the UV/Vis spectrum shifted to red because of the Cu defect states formed in the forbidden gap (Figure 15). The 7% Cu-CdS exhibited the best hydrogen production ability, with a rate of 1115 µmol h^−1^ g^−1^, which is almost 5.3 times higher than that of undoped CdS of 212 µmol h^−1^ g^−1^ (Figure 16A). Furthermore, a Cu-CdS/MoS_2_ composite can reach a hydrogen production rate of 10.18 mmol h^−1^ g^−1^, which is about 48 times higher than that of pure CdS (Figure 16B). MoS_2_ can provide a lot of active sites at the edge of its sheets, leading to improved hydrogen production. The photoelectric performances of photoelectrodes with composite structures are often better than those based on pure doping.

In addition to doping, the energy band structure of sulfides can also be modified by forming solid solutions with different precursors of sulfides. The energy band structure of solid solutions can be precisely controlled by changing the composition of different precursors. For example, the band gaps of Zn_x_Cd_1−x_S [72] and Cu_x_Ag_1−x_InS_2_ [73] can be precisely tuned by adjusting the composition of Zn/Cd and Cu/Ag, respectively. In this case, both of the tuned sulfides can respond to visible light, allowing for the efficient utilization of solar energy. Moreover, such solid solutions show better PEC performances than doped sulfides.

#### 3.2.2. Sulfide-Based Composites

**Figure 17:**
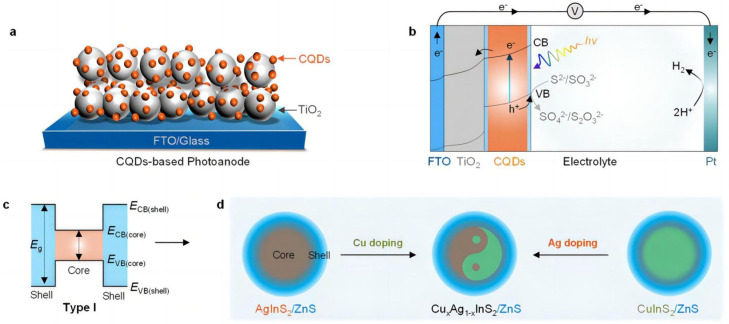
Manipulating the catalytic activity of photoanodes by modulating the structure of CQDs: (**a**) Schematic diagram for a CQD-based photoanode with a structure comprising glass|FTO|TiO_2_|CQDs. (**b**) Figure and predictable band alignment of a CQD-based photoanode photoelectrochemical cells. (**c**) The band structure of type-I CQDs. (**d**) Schematic illustration for band structure engineering and defect passivation of Cu-doped AgInS_2_/ZnS CQDs [73] (a detailed description of type-I heterojunctions is available in Section 3.3). Reproduced with permission from Guo, H., *ACS Omega*, published by American Chemical Society, 2022.

Like sulfides, sulfide-based composites have also attracted considerable attention [74]. Gao et al. [75] prepared ZnIn_2_S_4_ nanosheets on flexible graphite felt by using a hydrothermal method. They found that a photoelectrode with a 5 mm-thick graphite felt and ZnIn_2_S_4_ coating exhibited the best PEC properties compared to other photoelectrodes. Guo et al. [73] designed a series of Cu_x_Ag_1−x_InS_2_/ZnS colloidal quantum dots (CQDs) by the defect passivation of a ZnS shell and the incorporation of Cu ions to engineer its band structure. ZnS shell-assisted defect passivation suppressed charge carrier recombination because of the formation of the core/shell heterojunction interface, enhancing the performance of PEC devices with better charge separation and stability. Furthermore, Cu ion doping in AgInS_2_ CQDs results in a shift in the energy band of the quantum dots, which greatly promote the interface’s charge separation and transfer (Figure 17).

**Figure 18:**
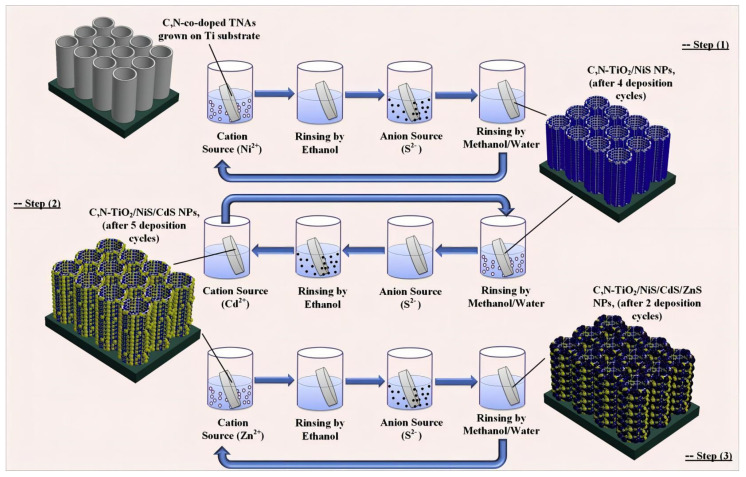
Schematic diagram illustrating the SILAR process for depositing NiS, CdS, and ZnS NPs on a C, N-co-doped TNA substrate [76]. Reproduced with permission from Mollavali, M., *International Journal of Hydrogen Energy*, published by Elsevier, 2018.

**Figure 19:**
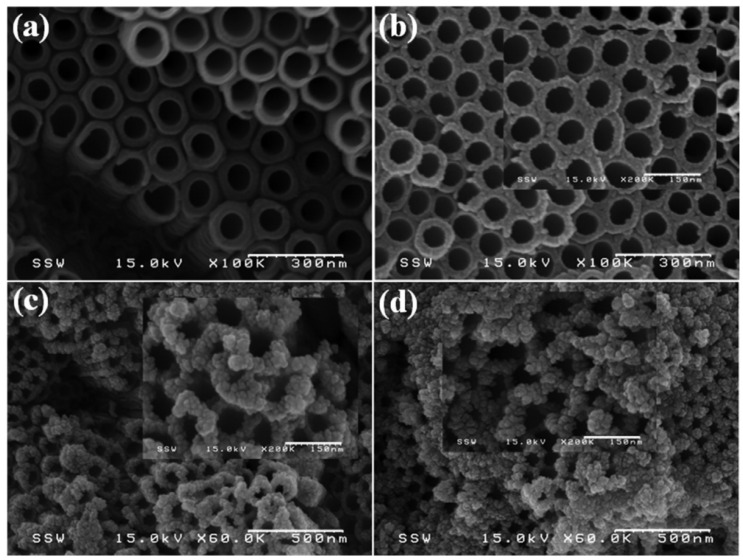
Top-view FE-SEM images for (**a**) C, N-co-doped TNAs; (**b**) C, N-TiO_2_/NiS; (**c**) C, N-TiO_2_/NiS/CdS; and (**d**) C, N-TiO_2_/NiS/CdS/ZnS electrodes [76]. Reproduced with permission from Mollavali, M., *International Journal of Hydrogen Energy*, published by Elsevier, 2018.

Mollavali [76] reported the composite structures of a variety of sulfides on Co-doped/modified TiO_2_. TiO_2_ nanostructures were first sensitized by nitrogen and carbon with a one-step low-cost anodic oxidation process, and then NiS/CdS/ZnS NPs were deposited on the surfaces of TiO_2_ nanostructures by successive ionic layer adsorption and using a successive ionic layer adsorption and reaction (SILAR) method at room temperature (Figure 18). These vertically aligned C, N-co-doped TiO_2_ nanotube arrays (TNAs) electrode provide a large surface area for the deposition of NPs, and they are also well-defined channels for efficient charge transport (Figure 19a). The NiS nanoparticles have small aggregation rather than large aggregation on the top of the TNAs (Figure 19b). Although some aggregates of the nanoparticles can be observed in the final photoanode due to the dense particle loading, the nanotubes remain predominantly open after all deposition steps, which is an important factor to maximize light absorbance and photoactivity, as well as electrolyte transport (Figure 19c,d). The optical properties of a C, N-TiO_2_/NiS/CdS/ZnS photoanode were enhanced due to a reduction in the recombination of electrons and holes, improving the surface carrier transfer rate and photogenerated carrier separation.

### 3.3. Graphite-Phase Carbon Nitride (g-C_3_N_4_)

In 2009, Wang [77] first reported that g-C_3_N_4_ can photocatalytically decompose water into hydrogen and oxygen with a sacrificial agent under visible light. The band gap of g-C_3_N_4_ is 2.7 eV. Its minimum conduction band and maximum valence band are −1.1 eV and +1.6 eV, respectively, which makes it possible to decompose water completely under visible light. It can be made by calcining common raw materials such as melamine, cyanuric chloride, and urea at high temperature. The C/N ratio of g-C_3_N_4_ varies depending on the calcination temperature [78].

#### 3.3.1. Doping

**Figure 20:**
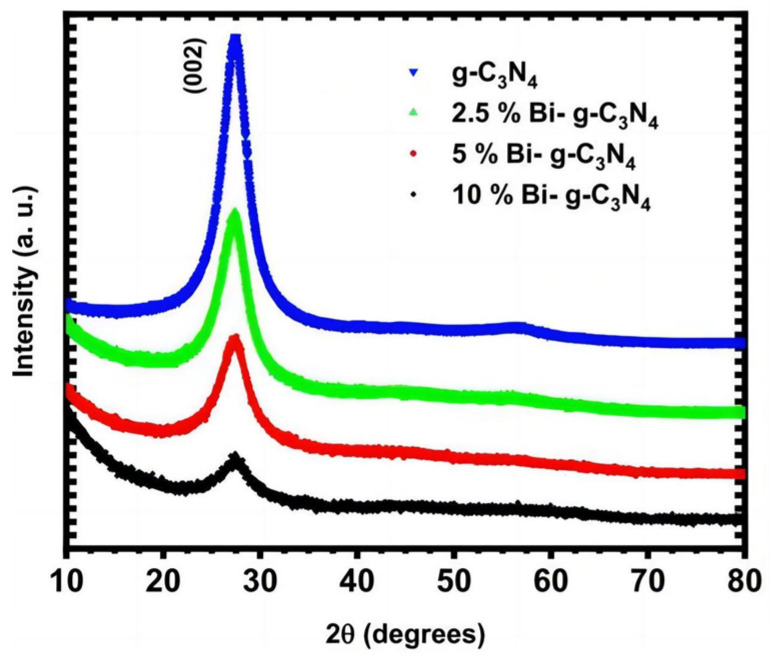
XRD patterns of g-C_3_N_4_ and a series of Bi-doped g-C_3_N_4_ samples [81]. Reproduced with permission from El Rouby, *Solar Energy*, published by Elsevier, 2020.

**Figure 21:**
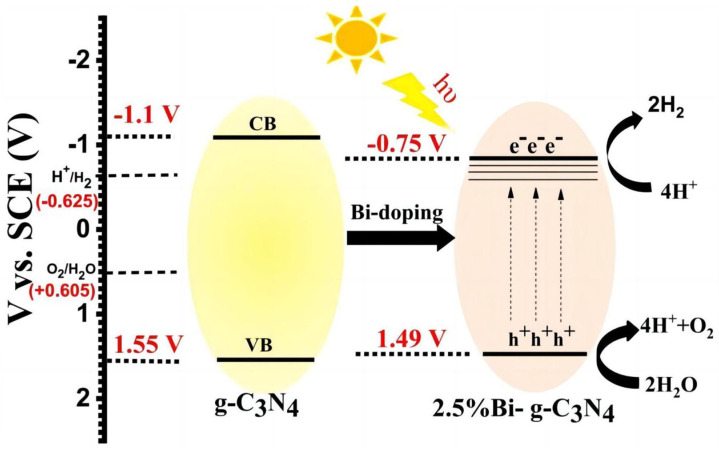
Schematic illustrations for the valence bands and the conduction bands of neat g-C_3_N_4_ and 2.5% Bi-g-C_3_N_4_ [81]. Reproduced with permission from El Rouby, *Solar Energy*, published by Elsevier, 2020.

**Figure 22:**
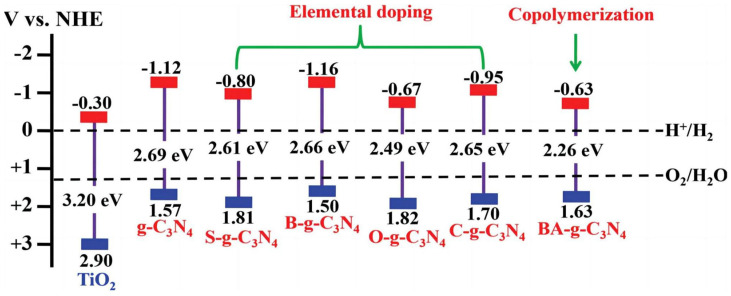
Schematic illustration of the band structures of typical g-C_3_N_4_ samples in comparison to TiO_2_ samples: g-C_3_N_4_, S-g-C_3_N_4_, B-g-C_3_N_4_, O-g-C_3_N_4_, C-g-C_3_N_4_, and BA-g-C_3_N_4_ [82]. Reproduced with permission from Cao, S., *Advanced Materials*, published by John Wiley and Sons, 2015.

Wang et al. [79] found that fluorine doping into g-C_3_N_4_ can form a C-F bond, narrowing its band gap and increasing its optical absorption range. Wu et al. [80] reported that phosphorus (P)-doped g-C_3_N_4_ synthesized by a simple sintering method exhibited 1.4 μA/cm^2^ of photocurrent at 1.2 V versus Ag/AgCl under near-IR light (>800 nm) irradiation. The introduction of P into g-C_3_N_4_ reduced its bandgap from 2.75 eV to 1.37 eV, in favor of a superior infrared light response. Meanwhile, the doping of P also improved the separation and transfer of photogenerated charges. In addition to non-metal doping, metal doping has also been extensively used to improve the PEC performance of g-C_3_N_4_. Rouby et al. [81] reported on the synthesis of an elongated g-C_3_N_4_ nanostructure which was fabricated by the direct pyrolysis of a supramolecular melamine precursor. The as-prepared material was used to host specific amounts of bismuth, a doping element used to adjust the band gap of the hosting matrix. XRD measurements confirmed the absence of bismuth oxide in the photoanode (Figure 20). The bandgap width was reduced due to the introduction of Bi in g-C_3_N_4_ (Figure 21). The 2.5% Bi-doped g-C_3_N_4_ photoelectrode was twice as efficient as pure g-C_3_N_4_ in terms of PEC water decomposition. The schematic diagram shown in Figure 22 shows the energy band structure of a typical element-doped and molecularly modified g-C_3_N_4_ sample.

#### 3.3.2. g-C_3_N_4_ Composites

**Figure 23:**
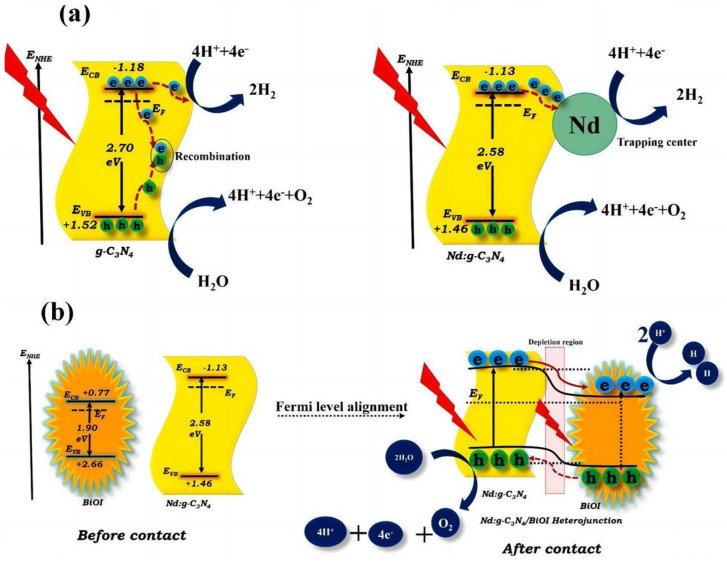
Schematic diagram of the PEC mechanism of (**a**) undoped and Nd-doped g-C_3_N_4_ samples and (**b**) a Nd-doped g-C_3_N_4_/BiOI heterostructure [88]. Reproduced with permission from Velusamy, P., *Applied Surface Science*, published by Elsevier, 2021.

Semiconductor heterojunctions can effectively promote the separation of photogenerated electron–hole pairs, thus enhancing the PEC activity of semiconductor materials. As a kind of flexible material, g-C_3_N_4_ is beneficial for closely compounding with other semiconductors. Available materials include metal oxides (ZnO [83,84], TiO_2_ [85,86], WO_3_ [87], BiIO [88], Al_2_O_3_ [89]), sulfides (CdS [90], MoS_2_ [91]), graphene [92], activated carbon [93], and noble metal Au [94]. Velusamy et al. [88] prepared a noble metal-free nano-het-erostructure of neodymium (Nd)-doped graphitic carbon nitride (g-C_3_N_4_) and bismuth oxyiodide (BiOI) by using a two-step thermal poly-condensation and hydrothermal method. The doping of Nd reduced the bandgap width of the photoelectrode, thereby increasing its response range to visible light. At the same time, the construction of heterojunctions can improve the transfer and suppress the recombination of photogenerated charges. The combination of the two methods improves the optical and electrical properties of the photoelectrode, leading to an enhancement in PEC performance (Figure 23).

## 4. Strategies to Improve the Efficiency of PEC Hydrogen Evolution

Based on the present understanding of the principle of PECs, we discussed the promising semiconductor materials and related experimental methods and means currently used in the PEC decomposition of water. PEC hydrogen evolution involves diversiform chemical and physical processes such as the preparation of a photoelectrode material, photon absorption, semiconductor excitation, and the separation and migration of electron–hole pairs. Considering these important chemical and physical processes involved in PECs, this section summarizes strategies that might be useful for improving the efficiency of PEC hydrogen production.

### 4.1. Morphological Control

The morphological properties of semiconductor photoelectrode materials, such as the thickness of film electrodes and the surface microstructure, have a very important influence on PEC efficiency. For example, the morphology of single-crystalline TiO_2_ NTs is beneficial for directional charge transport due to their nanotube confinement effect [95]. In one study, after size-controllable g-C_3_N_4_ quantum dots (QDs) were synthesized in situ and grafted onto TiO_2_ NTs, the unique morphology and structure efficiently inhibited self-gathering and the leaching of g-C_3_N_4_ QDs, leading to excellent PEC performance [95]. The reactions of oxidation and reduction occur only when the carrier charges migrate from the inside to the surface of the semiconductor photoelectrode or counter electrode. Thus, a considerable part of the carrier charges is likely to recombine before they reach the electrode surface due to the low carrier mobility or excessive thickness of the photoelectrode film. In this case, the thinner the photoelectrode film is, the faster the carrier charges can be transferred to the surface of the photoelectrode. However, excessive reduction of the thickness of the photoelectrode film will weaken the light absorption [96,97]. Therefore, a balance regarding the light harvest and fast transfer of the carrier charges should be achieved by optimizing the thickness of the photoelectrode film [98].

It is well known that crystal surfaces play an important role in the PEC activity of photoelectrodes. Compared with a stable anatase (101) crystal surface, a metastable (001) crystal surface has more unsaturated coordination surface dangling bonds. Yang [99] was the first to successfully synthesize anatase TiO_2_ micro crystals with an exposure ratio of (001) crystal plane up to 47% through the regulation of hydrofluoric acid (HF). This groundbreaking work led to an upsurge in the study of anatase (001) facet synthesis and related PEC properties. However, HF is highly corrosive. Thus, various fluorides have been used as additives to study the effect of stabilizing (001) crystal facets, including ammonium fluoride, ammonium hydrogen fluoride, sodium fluoride, or fluorine-containing surfactants [100]. BiVO_4_ film photoelectrodes composed of nanoplates with highly reactive exposed facets (001) also exhibit a photocurrent density more than five times higher than that of nanorods grown along the (100) direction. Similarly, WO_3_ nanomultilayers with highly exposed (002) facets (60%) exhibited much better PEC performances than WO_3_ nanorods with less exposed (002) facets (20%) [31].

### 4.2. Doping

Doping can create defect energy levels in the bandgap of a semiconductor, thus changing its energy band structure. On one hand, the defect energy levels usually offer a narrow sub-bandgap in the semiconductor, contributing to increasing the light absorption; on the other hand, the defect energy levels can form a charge capture center and thus increase the carrier life. In addition, doping can also regulate the electrical properties of semiconductors, such as carrier concentration and carrier mobility, enhance the conductivity and carrier mobility of semiconductors, and improve the efficiency of charge separation and transfer [101,102,103]. Therefore an optimal amount of doping should be determined for photoelectrode materials to achieve optimal PEC performance [104].

### 4.3. Heterojunctions

**Figure 24:**
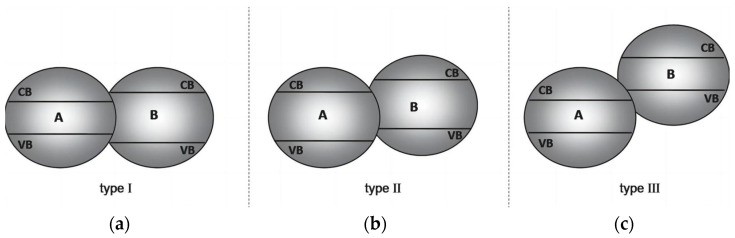
Different types of semiconductor heterojunctions. (**a**) Type-Ⅰ heterojunction, (**b**) type-II heterojunction, (**c**) type-Ⅲ heterojunction [107]. Reproduced with permission from Marschall, R., *Advanced Functional Materials*, published by John Wiley and Sons, 2013.

A heterojunction is the interfacial contact region of two different semiconductors; heterojunctions are mainly divided into types Ⅰ, Ⅱ, and Ⅲ. As shown in Figure 24a, in a type-Ⅰ heterojunction, both the conduction band (CB) and the valence band (VB) of semiconductor A are included in the bandgap of semiconductor B. The photogenerated electrons transfer from CB(B) to CB(A), while the photogenerated holes transfer from VB(B) to VB(A). All photogenerated charges are accumulated in semiconductor A, probably resulting in the recombination problem of charge carriers. In a type-Ⅱ heterojunction (Figure 24b), both the CB and the VB of semiconductor B are more negative than those of semiconductor A, meanwhile, the VB of semiconductor B is located in the bandgap of semiconductor A. In this case, the photogenerated electrons and holes are likely to accumulate in the CB (A) and VB(B), respectively, improving the efficiency of the separation of carrier charges, thus enhancing the PEC decomposition of water. Numerous composite photoelectrodes mentioned above employ this type-Ⅱ heterojunction structure. For example, the PEC performance of TiO_2_ can be significantly improved when coupled with Cu_2_O [105] and ZnO [106] to form type-Ⅱ heterojunctions. In a type-Ⅲ heterojunction (Figure 24c), the whole band position of semiconductor B is further offset to that of semiconductor A. Such arrangements of band positions are also called broken-gap situations.

**Figure 25:**
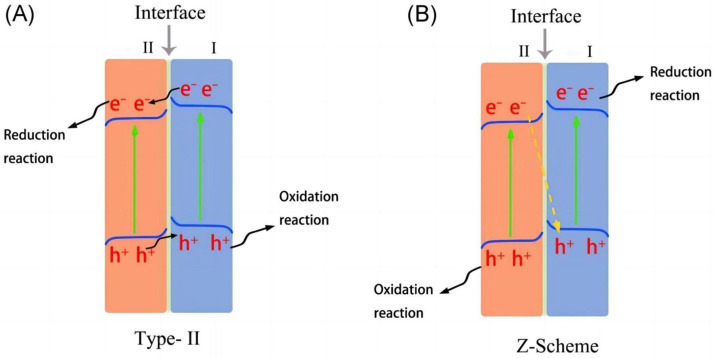
Schematic illustration of charge carrier transfer for (**A**) type-II and (**B**) Z-scheme heterojunctions [108]. Reproduced with permission from Li, J., *Carbon Energy*, published by John Wiley and Sons, 2022.

For type-II heterojunctions, despite the fact that they can effectively separate the charge, their oxidation–reduction abilities have not yet been maximized in this system. In contrast, Z-scheme heterojunctions can achieve strong oxidation–reduction abilities. As shown in Figure 25 [108], in Z-scheme heterojunctions, the photogenerated electrons in semiconductor II are inclined to combine with the photogenerated holes in semiconductor I. In this case, the photogenerated electrons and holes are likely to accumulate in the CB of semiconductor I and VB of semiconductor II, respectively. The structures of Z-scheme heterojunctions can both enhance the separation of charge carriers and retain the strong reduction and oxidation ability, thus improving the PEC performance of the photoelectrode.

### 4.4. Surface Modification

**Figure 26:**
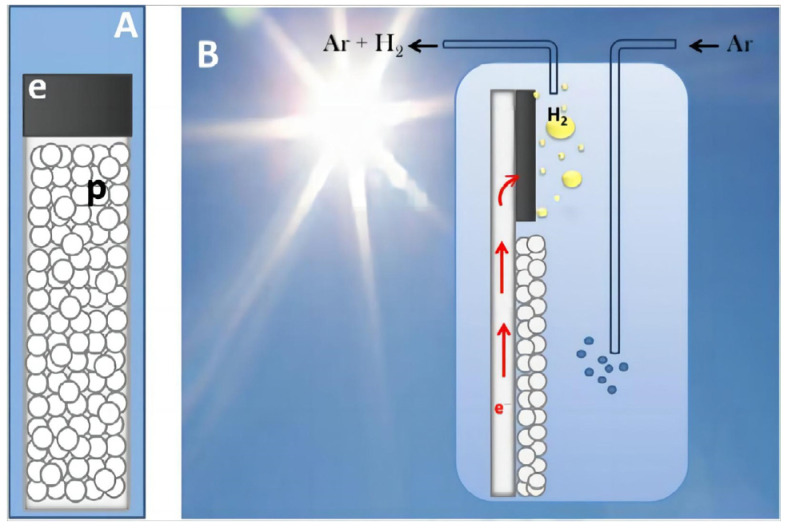
Illustration of a “PEC Leaf” (**A**) showing the area covered by the nanoparticulate photocatalyst (p), the area covered by the electrocatalyst (e), and a reactor producing hydrogen by employing a photocatalytic leaf (**B**) [109]. Reproduced with permission from Pop, L., *Applied Surface Science*, published by Elsevier, 2015.

Metals, especially noble metals (such as Pt) with large work functions (i.e., low Fermi levels) can effectively collect electrons and enjoy a low activation energy and overpotential for the hydrogen production reaction. Therefore, noble metals are often considered as suitable cocatalysts for PEC hydrogen production. Pop et al. [109] reported a “PEC Leaf” photoelectrode consisting of nano titanium dioxide particles as photocatalysts and a commercial carbon paste enriched with a small quantity of Pt NPs (0.0134 mg/cm^2^) as electrocatalysts. As shown in Figure 26, Pt can effectively enrich photogenerated electrons from the photoelectrode, greatly improving hydrogen production efficiency with the help of a sacrificial agent. It is worth noting that the loading amount of Pt directly affects the PEC performance of the sample, and the excessive loading of Pt will lead to a decrease in the PEC performance of the sample [110]. In addition, the hydrogen production performance of photoelectrodes can also be affected by the particle size and shape of the noble metal cocatalyst [111].

**Figure 27:**
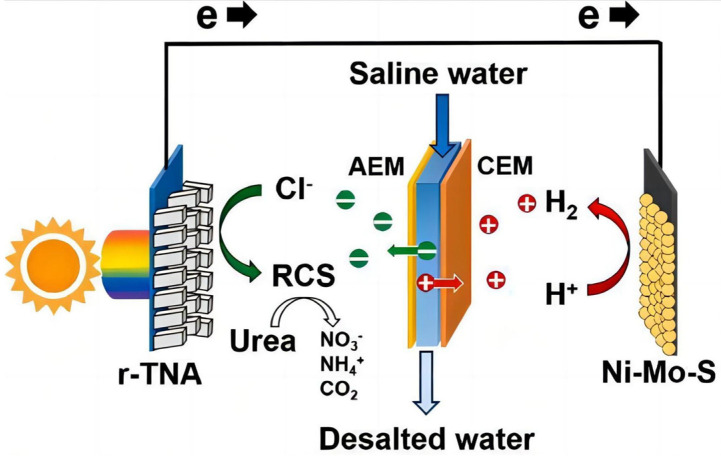
Illustration of a sunlight-driven ternary hybrid desalination device. The device consists of a thermochemically reduced TiO_2_ nanorod array (r-TNA) photoelectrode in the anode cell, saline water in the (middle) desalination cell, and a Ni-Mo-S (Ni_2_S_3_/MoS_2_) electrocatalyst in the cathode cell. The anode and desalination cells were separated by an anion exchange membrane (AEM), whereas the desalination cell and cathode cell were separated by a cation exchange membrane (CEM) [114]. Reproduced with permission from Kim, S., *Applied Catalysis B: Environmental*, published by Elsevier, 2021.

However, the high cost of noble metals limits their use on a large scale. So, some transition metals and oxides have been used as low-cost substitutes, such as NiO [112] and CuO [113]. Kim et al. [114] reported a ternary hybrid solar desalination process coupled with PEC water treatment and H_2_ production in a low-cost device with an oxide photoelectrode and transition sulfate cathode. The desalination of brackish water in the desalination cell is initiated via photoinduced charge generation with a thermochemically reduced TiO_2_ nanorod array photoanode. As shown in Figure 27, a three-cell device was designed to achieve hydrogen production and seawater desalination. The Ni-Mo-S composite catalyst greatly promoted the generation of hydrogen at the cathode, thereby improving the seawater desalination rate of the entire device.

## 5. Concluding Remarks

An overview of promising semiconductor materials for efficient PEC hydrogen production has been given. The general characteristics, preparation methods, and applications of metal oxides, sulfide materials, and g-C_3_N_4_, as well as their modifications and composites, have been covered, as summarized in Table 1. The benefits and drawbacks of the photoelectrode materials and their corresponding remedial schemes have also been discussed. An efficient PEC photoelectrode should possess an appropriate energy band structure, wide wavelength light absorption, effective carrier separation and transport, and high stability. Doping and modification are effective approaches for improving the PEC performance of hydrogen evolution and stability of photoelectrode materials. Particularly, constructing type-II and Z-scheme heterojunctions using multiple materials is also a good way to effectively promote the separation and transport of carrier charges. We hope a comprehensive understanding of the relationship between the structures and properties of semiconductor materials will benefit the precise control of semiconductor materials as photoelectrodes and thus promote the development of PEC hydrogen production.

## Figures and Tables

**Table 1:** A summary of promising photoelectrocatalysts with improved photoelectric performance for the hydrogen production.

Photoelectrocatalyst	Modification Strategy	Rate (µmol h^−1^ g^−1^)	Incident Light (nm)	Ref.
BiVO_4_/FTO	morphological control	150	UV-Vis	[22]
ZnO/Ag	surface modification	10	UV-Vis	[29]
W-TiO_2_ NTs	doping	24.97	UV-Vis	[36]
TiO_2_ nanotube arrays	morphological control	97	UV-Vis	[37]
Nb-TiO_2_/g-C_3_N_4_	doping/heterojunction	43.26	>400	[38]
Fe^3+^-TiO_2_	doping	12.5	>400	[41]
TiO_2_/WO_3_/FTO	heterojunction	210	UV-Vis	[43]
ITO/Cu_2_O/TiO_2_	heterojunction	12.15	UV-Vis	[48]
TiO_2_/Cu_2_O	protective layer/Ti^3+^	0.068	>420	[50]
N-BiVO_4_	doping	3.7	UV-Vis	[57]
Bi_2_S_3_/BiVO_4_	heterojunction	33.4	UV-Vis	[59]
MoS_2_/Cu_2_O	heterojunction	12.3	UV-Vis	[64]
TiO_2_-1 wt% Au@TiO_2_/Al_2_O_3_/Cu_2_O	heterojunction/surface modification	147	UV-Vis	[65]
MoS_2_/Cu-CdS	doping/heterojunction	1115	UV-Vis	[69]
CdS/ZnO	heterojunction	1008	>400	[71]
Cd_0.5_Zn_0.5_S	morphological control	14,440	>420	[72]
Pt/C-ZnIn_2_S_4_	morphological control/surface modification	1032.2	>400	[74]
FTO/P-g-C_3_N_4_	doping	1.27	>800	[80]
Nd-doped g-C_3_N_4_/BiOI	doping/heterojunction	288	>420	[88]
g-C_3_N_4_/reduction graphene oxide/nickel foam	heterojunction/morphological control	6000	>420	[92]
Pt-TiO_2_/C	morphological control/surface modification	300	UV-enhanced light	[109]
Ni-Mo-S/reduced titania nanorods	surface modification	40	UV-Vis	[114]

## Data Availability

Not applicable.
